# Intestine microbiota and SCFAs response in naturally *Cryptosporidium*-infected plateau yaks

**DOI:** 10.3389/fcimb.2023.1105126

**Published:** 2023-03-01

**Authors:** Hailong Dong, Xiushuang Chen, Xiaoxiao Zhao, Chenxi Zhao, Khalid Mehmood, Muhammad Fakhar-e-Alam Kulyar, Zeeshan Ahmad Bhutta, Jiangyong Zeng, Shah Nawaz, Qingxia Wu, Kun Li

**Affiliations:** ^1^ Key Laboratory of Clinical Veterinary Medicine in Tibet, Tibet Agriculture and Animal Husbandry College, Linzhi, Tibet, China; ^2^ Institute of Traditional Chinese Veterinary Medicine, College of Veterinary Medicine, Nanjing Agricultural University, Nanjing, China; ^3^ MOE Joint International Research Laboratory of Animal Health and Food Safety, College of Veterinary Medicine, Nanjing Agricultural University, Nanjing, China; ^4^ Department of Clinical Medicine and Surgery, Faculty of Veterinary and Animal Sciences, The Islamia University of Bahawalpur, Bahawalpur, Pakistan; ^5^ College of Veterinary Medicine, Huazhong Agricultural University, Wuhan, China; ^6^ Laboratory of Biochemistry and Immunology, College of Veterinary Medicine, Chungbuk National University, Cheongju, Chungbuk, Republic of Korea; ^7^ Institute of Animal Husbandry and Veterinary Medicine, Tibet Academy of Agricultural and Animal Husbandry Sciences, Lhasa, China; ^8^ Department of Anatomy, Faculty of Veterinary Science, University of Agriculture, Faisalabad, Pakistan

**Keywords:** *Cryptosporidium*, yaks, diarrhea, microbiota, SCFAs

## Abstract

Diarrhea is a severe bovine disease, globally prevalent in farm animals with a decrease in milk production and a low fertility rate. *Cryptosporidium* spp. are important zoonotic agents of bovine diarrhea. However, little is known about microbiota and short-chain fatty acids (SCFAs) changes in yaks infected with *Cryptosporidium* spp. Therefore, we performed 16S rRNA sequencing and detected the concentrations of SCFAs in *Cryptosporidium*-infected yaks. Results showed that over 80,000 raw and 70,000 filtered sequences were prevalent in yak samples. Shannon (*p*<0.01) and Simpson (*p*<0.01) were both significantly higher in *Cryptosporidium-*infected yaks. A total of 1072 amplicon sequence variants were shared in healthy and infected yaks. There were 11 phyla and 58 genera that differ significantly between the two yak groups. A total of 235 enzymes with a significant difference in abundance (*p*<0.001) were found between healthy and infected yaks. KEGG L3 analysis discovered that the abundance of 43 pathways was significantly higher, while 49 pathways were significantly lower in *Cryptosporidium*-infected yaks. The concentration of acetic acid (*p*<0.05), propionic acid (*p*<0.05), isobutyric acid (*p*<0.05), butyric acid (*p*<0.05), and isovaleric acid was noticeably lower in infected yaks, respectively. The findings of the study revealed that *Cryptosporidium* infection causes gut dysbiosis and results in a significant drop in the SCFAs concentrations in yaks with severe diarrhea, which may give new insights regarding the prevention and treatment of diarrhea in livestock.

## Introduction

The long-haired ruminant yak is a plateau bovine species living in the 3000-5000 m high-altitude regions and is mostly found on the Qinghai Tibet plateau (Li et al., 2022a). Diarrhea is a serious bovine problem detected globally in livestock farms associated with a decrease in fertility rate and milk production, especially neonatal diarrhea is usually found with high morbidity and mortality (Han et al., 2017; Li et al., 2019a; Lan et al., 2021)

Previously, studies revealed that diarrhea contributed to more than 50% of calf mortality in Canada (Smith et al., 2014), and affected 19% of the cattle population in the USA (Smulski et al., 2020), which indeed was the cause of huge economic detriment. Like other bovine animals, diarrhea has been commonly reported in yaks (Diao et al., 2020; Cui et al., 2022; Li et al., 2022a). There have been many biological factors which are associated for diarrhea and leading cause of death in calves (Kim et al., 2021). Many pathogens like bovine viral diarrhea virus, Noroviruses, *Escherichia coli*, *Salmonella* spp., and *Cryptosporidium* spp. have been commonly observed in infected cattle (Meganck et al., 2014; Cui et al., 2022). Among others, *Cryptosporidium* spp. are important zoonotic protozoa infecting various animal species (Li et al., 2019b; Kandeel et al., 2022), and are also generally recognized as the primary agent of cattle diarrhea (Li et al., 2019a; Li et al., 2019b). A previous study reported that the infection of *Cryptosporidium* spp. was an important issue in UK and Scotland (Smith et al., 2014). As yaks and cattle species are economically important for native herdsmen in China (Cheng et al., 2022), infectious diseases like those caused by *Cryptosporidium* spp. may not only affect animal health but are also potential threats leading to public health concerns.

Intestine microbiota is composed of millions of complex and diverse microorganisms, which contribute greatly to host health, nutrition absorption, host metabolism, and immunological development (Zeineldin et al., 2018). Previous studies demonstrated that this bacteria was related to various diseases like Type 2 diabetes (Martinez-Lopez et al., 2022), acute pancreatitis (Mei et al., 2022), obesity (Salazar et al., 2022), and diarrhea (Han et al., 2017; Zeineldin et al., 2018; Li et al., 2022b). Short-chain fatty acids are metabolic products of microbiota, which contribute to the cellular metabolism of the host (Bachem et al., 2019), regulating immune function and suppressing inflammatory reactions (Abdalkareem Jasim et al., 2022). In our previous study, we observed prominent changes in intestinal microbiota in a horse infected with *Cryptosporidium* spp. (Wang et al., 2022). However, scarce information is available about microbiota and SCFAs changes in plateau yaks infected with *Cryptosporidium* spp. Therefore, this study was conducted to explore intestinal microbiota and SCFAs response to natural *Cryptosporidium* infection in plateau yaks.

## Materials and methods

### Samples

Fecal samples (n=40) were collected from free-ranged yaks in Xining, Qinghai (North latitude 31˚36´-39˚19´, east longitude 89˚35´-103˚04´) and examined for *Cryptosporidium* spp. by employing nested PCR (Chen et al., 2022) and positive samples were saved for further analysis. In this study, all the *Cryptosporidium* spp. positive samples (n=4) with equal number of negative samples (n=4) were sequenced and divided into infected (INF) and healthy (H) groups, respectively.

### DNA extraction and PCR amplification

The extraction of total genomic DNA was performed by utilizing a commercial TIANamp Stool DNA Kit (Tiangen Biotech (Beijing) Co., Ltd, China) according to the product’s specifications. Fecal DNA concentration, purification, and quality examination were performed through NanoDrop 2000 UV-Vis spectrophotometer (Thermo Scientific, USA) and 1.2% agarose gel electrophoresis, respectively. Then the hypervariable regions of bacterial 16S rRNA gene (V3-V4) were amplified using primers 338F and 806R as described in a previous study (Wang et al., 2019). All PCR products were individually subjected to agarose gel electrophoresis, gel extraction, and purification using the PureLink™ PCR Purification kit (Invitrogen™, USA). Finally, the purified DNA products were quantified by piloting QuantiFluor™-ST as guided by the instruction manual (Promega, USA).

### Library construction, Illumina miSeq sequencing, and bioinformatics analysis

Library construction was carried out by employing commercial Hieff NGS^®^ OnePot II DNA Library Prep Kit for Illumina^®^ (Yeasen, China) according to the product’s instructions, and sequenced through the Illumina NovaSeq platform (Illumina, San Diego, USA). Quality control of sequencing data was performed by employing QIIME2 (https://docs.qiime2.org/2019.1/) to generate amplicon sequence variant (ASV) (Callahan et al., 2016) and taxonomy table (Bokulich et al., 2018). Analysis of variance was performed using ANCOM (Analysis of Composition of Microbiomes), One-way ANOVA, Kruskal Wallis, LEfSe (LDA (Linear Discriminant Analysis) score >2), DEseq2 (*p*<0.05 and log2 (FoldChange) > 2), clustering heatmap (with Z-score > 0.5 or < -0.5) and evolutionary tree (*p*<0.05) methods to reveal differences in bacterial abundance among yak samples (Segata et al., 2011; Love et al., 2014; Mandal et al., 2015). Microbial alpha diversities analyses were performed through QIIME2 by calculating indices including observed OTUs, Chao1, Shannon, and Faith’s. Microbial beta diversities of principal coordinate analysis (PCoA), nonmetric multidimensional scaling (NMDS) (Vazquez-Baeza et al., 2013), and partial least squares discriminant analysis (PLS-DA) were carried out to explore the structural variation of microbial communities across yak samples. The evolutionary relation tree was constructed by using ggtree in R package.

### Function analysis

The potential KEGG Ortholog (KO) functional profiles of yak microbiota was predicted with PICRUSt (Langille et al., 2013) by annotating with MetaCyc and ENZYME database. One-way ANOVA was used to analyze the data, while Duncan test was used as *post-hoc* test to measure the individual differences in microbial function between the yak groups with a *p*<0.05 as statistically significant.

### SCFAs detection

The concentrations of SCFAs in fecal samples were detected by employing GC-MS (Hsu et al., 2019; Zhang et al., 2019), and the differences between yak groups were explored *via* t-test.

### Statistical analysis

The differences between different yak groups were calculated by the chi-square test piloting IBM SPSS Statistics (SPSS 22.0). *P* values < 0.05 were considered as statistically significant.

## Results

### Analysis of 16S rDNA sequencing data

**Figure 1:**
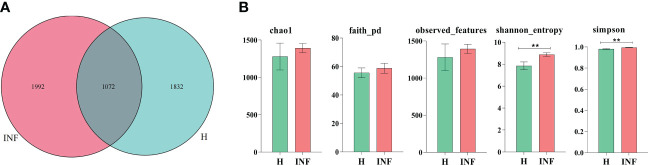
ASV venn map and Alpha diversity index analysis. **(A)** Venn map, **(B)** Alpha diversity index. ** refers to significance level, *p*<0.05.

In the current study, over 80,000 raw and 70,000 filtered sequences were obtained in yak samples. The non-chimeric sequences ranged from 62,133 to 73,453 in healthy yaks, and 68,173 to 74,350 in infected yaks (**Table 1**). There were a total of 1072 shared ASVs between the healthy (group H) and infected (group INF) groups. (**Figure 1A**). Alpha diversity index analysis showed that there was no significant difference in chao1, faith, and observed features between group H and INF, respectively. Shannon (*p*<0.01) and Simpson (*p*<0.01) were both significantly higher in group INF than in group H (**Figure 1B**).

**Table 1:** The sequence data statistic analysis.

Samples	input	filtered	percentage of input passed filter	denoised	merged	percentage of input merged	non- chimeric	percentage of input non-chimeric
H1	93773	86437	92.18	82758	76547	81.63	73453	78.33
H2	88135	81010	91.92	77501	71312	80.91	67743	76.86
H3	91889	85557	93.11	82297	76216	82.94	72913	79.35
H4	80446	74466	92.57	71353	65482	81.4	62133	77.24
INF1	89759	83135	92.62	78940	72488	80.76	68173	75.95
INF2	92942	86291	92.84	82357	75217	80.93	71848	77.3
INF3	89810	83187	92.63	80203	75295	83.84	74350	82.79
INF4	88245	81820	92.72	78721	73786	83.61	71099	80.57

### Grouping of yak microbiota in different taxa

**Figure 2:**
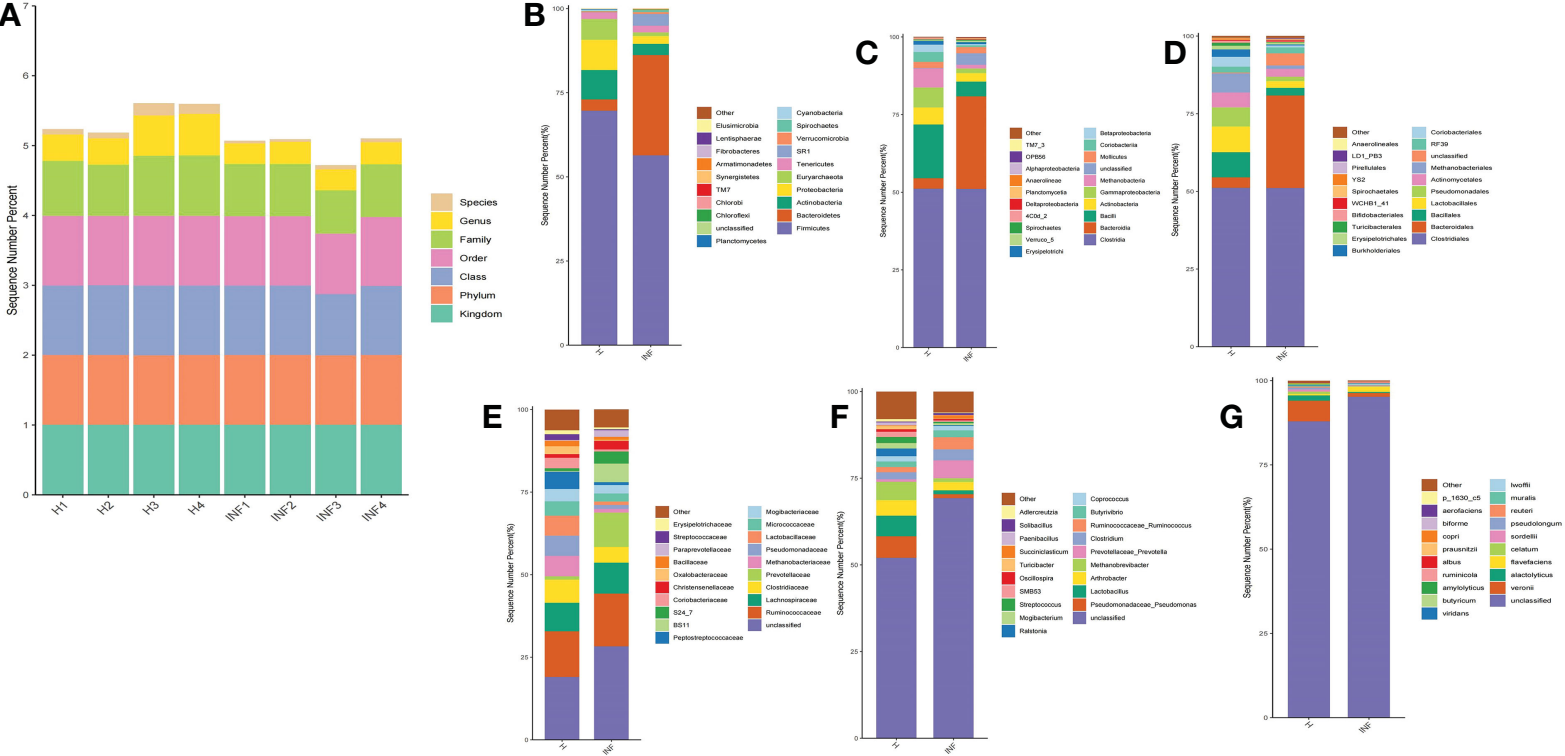
Statistical analysis of yak microbiota in different taxa. **(A)** Sequence percentages in different taxa, **(B)** Phylum, **(C)** Class, **(D)** Order, **(E)** Family, **(F)** Genus, **(G)** Species.

The sequence percentage in different taxa of group H and INF is shown in **Figure 2A**. At the phylum level, the dominant phyla were *Firmicutes* (69.61%), *Proteobacteria* (8.97%), and *Actinobacteria* (8.72%) in group H, while *Firmicutes* (56.38%) and *Bacteroidetes* (29.83%) were the main phyla in group INF (**Figure 2B**). At the class level, *Clostridia* (51.13%) and *Bacilli* (17.28%) were the primary classes in healthy yaks, while *Clostridia* (51.13%) and *Bacteroidia* (29.83%) were the major classes in infected yaks (**Figure 2C**). At the order level, *Clostridiales* (51.13%), *Lactobacillales* (8.20%), and *Bacillales* (8.10%) were the primary orders in healthy yaks, while *Clostridiales* (51.04%) and *Bacteroides* (29.83%) were the main orders in infected yaks (**Figure 2D**). At the family level, the main families were unclassified, *Ruminococcaceae* and *Lachnospiraceae* in groups H and INF (**Figure 2E**). At the genus level, unclassified (52.06%), *Pseudomonadaceae Pseudomonas* (6.13%), and *Lactobacillus* (6.00%) were the dominating genera in healthy yaks, while unclassified (69.25%), *Prevotellaceae Prevotella* (5.13%) and *Arthrobacter* (2.45%) were the main genera in infected yaks (**Figure 2F**). At the species level, the main bacteria in group H were unclassified (87.85%), *Veronii* (6.11%), and *Alactolyticus* (1.66%), while unclassified (95.15%), *Flavefaciens* (1.50%) and *Veronii* (1.12%) were the main bacteria in group INF (**Figure 2G**).

### Shifts of yak microbiota infected by *Cryptosporidium*

**Figure 3:**
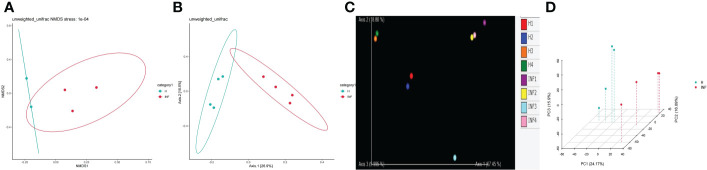
Beta diversity analysis between yak groups. **(A)** NMDS, **(B)** PCoA, **(C)** Qiime 2β, **(D)** PCA.

**Figure 4:**
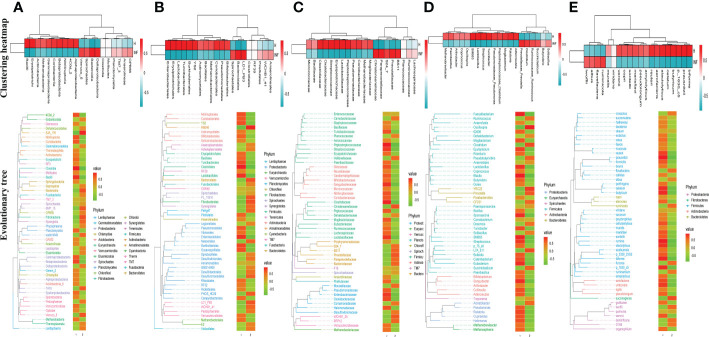
Clustering heatmap and evolutionary tree with heat map analysis of yak microbiota in different taxa. **(A)** Class, **(B)** Order, **(C)** Family, **(D)** Genus, **(E)** Species.

To reveal the microbiota difference between healthy and infected yaks, beta diversity analysis was carried out through NMDS, PCoA, Qiime 2β, and PCA analysis. The results showed a huge difference in composition and structure between samples from group H and group INF animals (**Figure 3**). To explore the microbiota changes caused by *Cryptosporidium* in different taxa, a clustering heatmap (top 20 abundance) and evolutionary tree (top 50 abundance) with heat map analysis were plotted. The results revealed that at the order level, infected yaks showed an abundance of *Bacteroidia* and *Deltaproteobacteria*, while healthy animals showed abundance of *Bacilli*, *Erysipelotrichi*, *Betaproteobacteria*, *Alphaproteobacteria*, and *Nitriliruptoria* as expressed in the clustering heatmap. The evolutionary tree also showed an obvious abundance difference in *Betaproteobacteria*, *Fibrobacteria*, SJA_176, 4C0d_2, *Nitriliruptoria*, *Clostridia*, and *Bacilli* between groups H and INF (**Figure 4A**). At the order level, the clustering heatmap revealed significant differences in the abundance of *Bacteroidales*, *Lactobacillales*, *Burkholderiales*, *Erysipelotrichales*, YS2, *Turicibacterales* and *Enterobacteriales* between healthy and infected animals. Evolutionary tree detected remarkable differences in the abundance of *Oceanospirillales*, *Burkholderiales*, *Enterobacteriales*, *Fibrobacterales*, *Turicibacterales*, RB046, YS2, *Nitriliruptorales*, *Clostridiales* and *Lactobacillales* between healthy and infected animals (**Figure 4B**). At the family level, there was a noteworthy difference of *Clostridiaceae*, *Prevotellaceae*, *Lactobacillaceae*, *Peptostreptococcaceae*, BS11, *Christensenellaceae*, *Oxalobacteraceae*, *Paraprevotellaceae*, *Streptococcaceae* and *Erysipelotrichaceae* between groups H and INF as revealed by the clustering heatmap. Evolutionary tree analysis showed a clear difference of *Halomonadaceae*, *Oxalobacteraceae*, *Enterobacteriaceae*, *Streptococcaceae*, *Peptostreptococcaceae*, *Turicibacteraceae*, *Dietziaceae*, *Sanguibacteraceae*, *Nitriliruptoraceae*, *Christensenellaceae*, *Clostridiaceae* and *Lactobacillaceae* between healthy and infected yaks (**Figure 4C**). At the genus level, interesting difference of *Lactobacillus*, *Prevotellaceae*_*Prevotella*, *Ralstonia*, *Streptococcus*, SMB53, *Turicibacter*, and *Adlercreutzia* was found between the two yak groups. Evolutionary tree analysis demonstrated that the abundance of *Halomonadaceae*, *Oxalobacteraceae*, *Streptococcaceae*, *Clostridiaceae*, *Turicibacteraceae*, *Planococcaceae*, *Erysipelotrichaceae*, *Sanguibacteraceae*, *Coriobacteriaceae*, *Paraprevotellaceae*, *Ruminococcaceae*, *Lachnospiraceae*, *Clostridiaceae*, *Lactobacillaceae* and *Lachnospiraceae* were significantly different between the two yak groups (**Figure 4D**). At the species level, the abundance of *alactolyticus*, *celatum*, *reuteri*, *butyricum*, *ruminicola*, *prausnitzii*, *biforme*, p_1630_c5, and *aerofaciens* were noticeably different in groups H and INF. Evolutionary tree analysis uncovered that the abundance of *alactolyticus*, *ruminis*, p_1630_c5, *biforme*, *umbonata*, *aerofaciens*, *prausnitzii*, *butyricum*, *celatum* and *reuteri* were significantly different between healthy and infected animals (**Figure 4E**).

**Figure 5:**
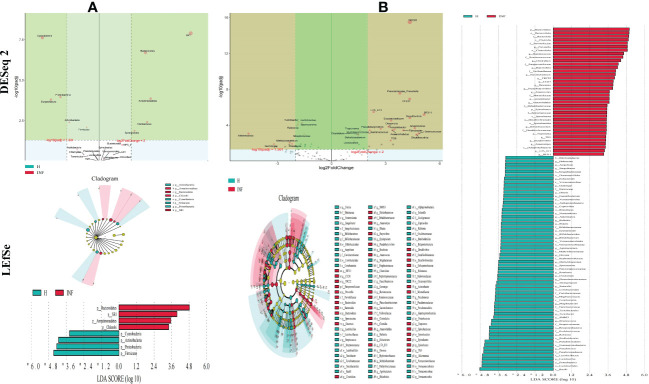
*Cryptosporidium* infection changes microbiota in different taxa through DESeq 2 volcano plot and LEFSe analysis. **(A)** Phylum, **(B)** Genus.

To further uncover the marker bacteria between healthy and *Cryptosporidium*-infected yaks, we performed one-way ANOVA and Kruskal Wallis tests to determine the significance of the difference and depicted results by DESeq 2 volcano diagram and LEfSe chart, respectively. Results showed that at the phylum level, the abundance of SR1 (*p*<0.0001), *Bacteroidetes* (*p*<0.0001), *Armatimonadetes* (*p*<0.0001), *Fibrobacteres* (*p*<0.01), and *Synergistetes* (*p*<0.01) were visibly higher in infected yaks, while *Cyanobacteria* (*p*<0.0001), *Proteobacteria* (*p*<0.0001), *Armatimonadetes* (*p*<0.0001), *Euryarchaeota* (*p*<0.0001), *Actinobacteria* (*p*<0.01), *Firmicutes* (*p*<0.01), and *Elusimicrobia* (*p*<0.05) were significantly lower (**Figure 5A**). At the genus level, the abundance of YRC22 (*p*<0.0001), *Prevotellaceae*_*Prevotella* (*p*<0.0001), CF231 (*p*<0.0001), L7A_E11 (*p*<0.0001), BF311 (*p*<0.0001), *Desulfovibrio* (*p*<0.0001), *Succiniclasticum* (*p*<0.0001), *Desemzia* (*p*<0.0001), *Anaerovorax* (*p*<0.0001), *Pseudobutyrivibrio* (*p*<0.0001), *Acinetobacter* (*p*<0.0001), *Fibrobacter* (*p*<0.0001), *Ruminococcaceae*_*Ruminococcus* (*p*<0.0001), *Anaerorhabdus* (*p*<0.0001), *Treponema* (*p*<0.0001), *Selenomonas* (*p*<0.001), *Clostridium* (*p*<0.001), *Shuttleworthia* (*p*<0.001), *Dehalobacterium* (*p*<0.001), TG5 (*p*<0.01), unclassified (*p*<0.01), *Anaerostipes* (*p*<0.01), *Syntrophomonas* (*p*<0.01), *Brachymonas* (*p*<0.01), *Pyramidobacter* (*p*<0.01), SHD_231 (*p*<0.05), *Butyrivibrio* (p<0.05), *Desulfobulbus* (p<0.05), RFN20 (p<0.05), and *Anaerofustis* (*p*<0.05) were significantly higher in infected yaks, while *Turicibacter* (*p*<0.0001), *Lactobacillus* (*p*<0.0001), *Sporosarcina* (*p*<0.0001), *Ralstonia* (*p*<0.0001), *Akkermansia* (*p*<0.001), *Streptococcus* (*p*<0.001), *Methylobacterium* (*p*<0.01), *Adlercreutzia* (*p*<0.01), *Faecalibacterium* (p<0.01), *Roseburia* (*p*<0.01), *Paenibacillus* (*p*<0.01), *Methanosphaera* (*p*<0.01), *Pseudomonadaceae*_*Pseudomonas* (*p*<0.01), *Peptostreptococcaceae*_*Clostridium* (*p*<0.01), *Slackia* (*p*<0.01), *Cupriavidus* (*p*<0.01), *Halomonas* (*p*<0.01), *Gemmiger* (*p*<0.01), *Dietzia* (*p*<0.01), *Blautia* (*p*<0.05), *Agrobacterium* (*p*<0.05), *Nesterenkonia* (*p*<0.05), *Sanguibacter* (*p*<0.05), *Phascolarctobacterium* (*p*<0.05), *Actinomycetospora* (*p*<0.05), *Bifidobacterium* (*p*<0.05), SMB53 (*p*<0.05), and *Dorea* (*p*<0.05) were significantly lower in infected animals (**Figure 5B**).

### *Cryptosporidium* infection potentially affected the microbiota function of yaks

**Figure 6:**
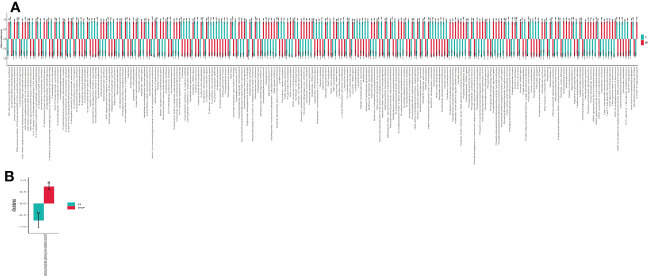
*Cryptosporidium* infection affected enzyme and MetaCys pathway abundance of yaks. **(A)** Enzyme (*p*<0.001), **(B)** MetaCys (*p*<0.05). "a, b" are showing significance relation.

**Figure 7:**
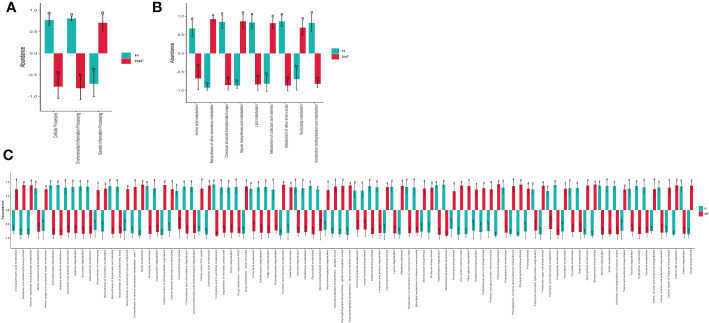
*Cryptosporidium* infection potentially affected the microbiota function of yaks. **(A)** KEGG L1 (*p*<0.05), **(B)** KEGG L2 (*p*<0.05), **(C)** KEGG L3 (*p*<0.05). "a, b" are showing significance relation.

The prediction of yaks’ microbiota function was carried out by PICRUSt2, and the functional difference between yaks was explored by using one-way ANOVA and Duncan test through R language as previously reported (Zhai et al., 2020). A total of 235 enzymes with a significant difference in abundance (*p*<0.001) were found between healthy and infected yaks, with 119 higher and 116 lower abundance enzymes in INF yaks (**Figure 6A**). Only one different MetaCys pathway of pentose phosphate pathway (non-oxidative branch) was found between the two yak groups (**Figure 6B**). KEGG L1 analysis found that the abundance of genetic information processing was prominently higher in infected yaks, while cellular processes and environmental information processing were significantly lower (**Figure 7A**). KEGG L2 analysis revealed that the abundance of biosynthesis of other secondary metabolites, glycan biosynthesis, and metabolism, metabolism of cofactors and vitamins, and nucleotide metabolism were remarkably higher in INF yaks, while amino acid metabolism, chemical structure transformation maps, lipid metabolism, metabolism of other amino acids, xenobiotics biodegradation, and metabolism were conspicuously lower (**Figure 7B**). KEGG L3 analysis discovered that the abundance of 43 pathways was significantly higher in INF yaks, while 49 pathways were significantly lower (**Figure 7C**).

### *Cryptosporidium* infection decreased the concentration of SCFAs in yaks

**Figure 8:**
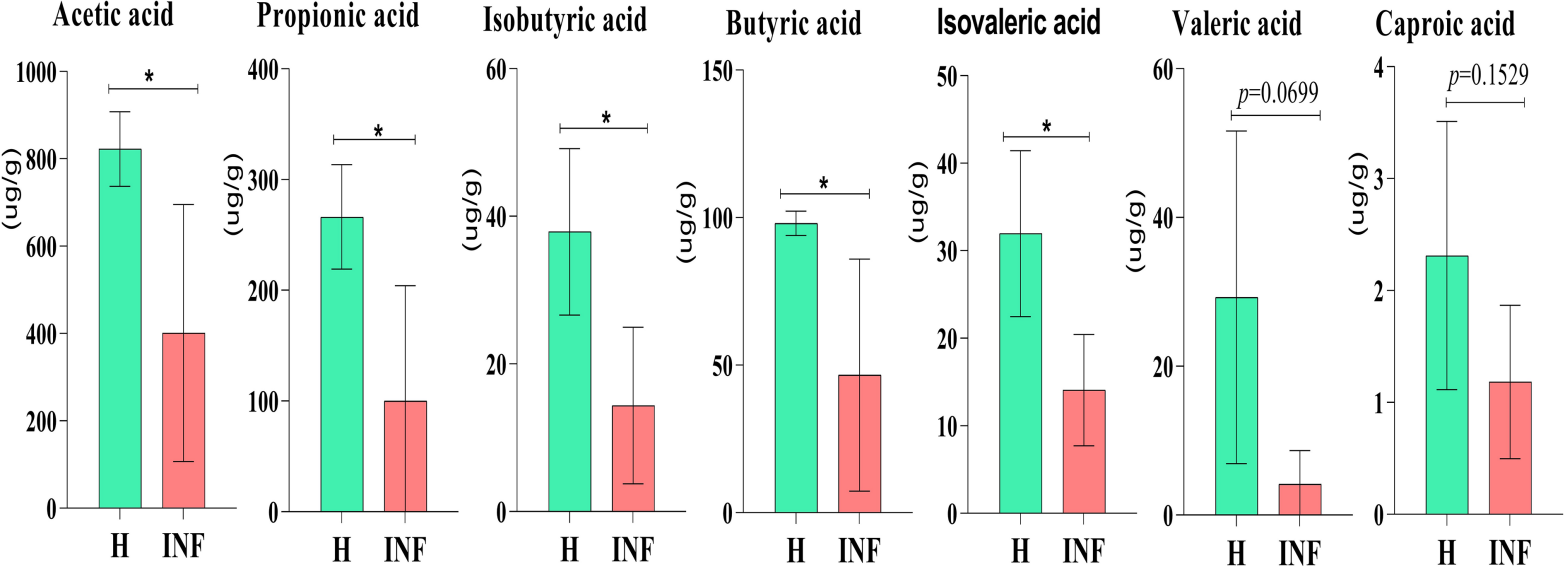
Concentration of SCFAs in yaks. Significance is presented as *p < 0.05; data are presented as the mean ± SEM (n = 4).

The concentration of acetic acid (*p*<0.05), propionic acid (*p*<0.05), isobutyric acid (*p*<0.05), butyric acid (*p*<0.05) and isovaleric acid was significantly lower in infected yaks, respectively, while there was no significant difference of valeric acid and caproic acid between H and INF groups (**Figure 8**).

## Discussion

Cattle diarrhea is still an important worldwide issue on farms, despite observing advanced preventive measures such as herd management, animal facilities and care, feeding and nutrition, and timely medication (Wei et al., 2021). The infectious *Cryptosporidium* was one of the main causative agents of diarrhea with limited available effective treatments (Li et al., 2019a). The harsh climatic conditions with heavy snowfall in the long frigid season (from October to May, with average temperature −15 to −5°C) didn’t permit collection of many samples in the Plateau region. Also, very few positive samples (n=4) were observed out of total collected samples (n=40) in the present study. However, a prevalence as low as 1.3% of Cryptosporidium spp. positive samples has been reported in yaks in China region (Li et al., 2020). Moreover, despite the harsh climatic conditions and the low number of positive samples available for analysis, this number was above the minimum required for high throughput sequencing, and validation of changes of the microbiota (Ray et al., 2019). In the current study, we performed 16S rDNA sequencing of fecal samples collected from healthy and *Cryptosporidium-*infected yaks. Results showed that *Cryptosporidium* infection increased the alpha diversity index of Shannon (*p*<0.01) and Simpson (*p*<0.01) (**Figure 1B**), which demonstrated the increased microbiota complexity of infected animals. The current results are in line with our previous results found in *Cryptosporidium-*infected horses (Wang et al., 2022). Beta diversity analysis through NMDS, PCoA, Qiime 2β, and PCA analysis revealed microbiota differences between healthy and infected yaks (**Figure 3**), which were confirmed by comparing the dominating gut microbiota in different taxa (**Figures 2**, **4**). Then we explored the significantly different bacteria between the H and INF groups through DESeq 2 volcano diagram and LEfSe chart analysis. The results showed that a total of 11 phyla and 58 genera were significantly different (**Figure 5**), which is in accordance with the previously reported results in a study conducted on infected people and horses (Chappell et al., 2016; Wang et al., 2022). The increased genera in yaks were in line with previous studies that found a higher abundance of *Desulfovibrio* and *Butyrivibrio* in colitis patients (Berry and Reinisch, 2013; Gryaznova et al., 2021), *Prevotellaceae*_*Prevotella* in diarrheic pigs (Yang et al., 2017), *Anaerovorax* in slow growth performers in nursery pigs (Zhai et al., 2020), *Succiniclasticum* in LPS induced dual-flow continuous culture system (Dai et al., 2019), *Pseudobutyrivibrio* in chronic kidney people (Wu et al., 2020), *Anaerorhabdus* in pulmonary fibrosis persons (Tong et al., 2019), *Selenomonas* in gastric cancer patients (Zhang et al., 2021), *Anaerostipes* in diabetic nephropathy patients (Du et al., 2021), *Pyramidobacter* in endoscopic sphincterotomy surgery gallstone patients (Shen et al., 2021), and *Anaerofustis* in Alzheimer people (Hou et al., 2021). The genus of *Acinetobacter* is an underrated food-borne pathogen (Amorim and Nascimento, 2017). A previous study found *Acinetobacter* in acute diarrhea of children (Polanco and Manzi, 2008). The genus of *Treponema* is the main pathogen in bovine dermatitis (Mamuad et al., 2020), *Clostridia* are clinical species and some of them may cause severe infections like colitis (Sanchez Ramos and Rodloff, 2018). Those increased genera may have contributed greatly to diarrhea caused by *Cryptosporidium.* The lower abundance of genera in yaks was in accordance with the results revealing *Turicibacter* and *Lactobacillus* in *Salmonella-*infected pigs (Garrido et al., 2021), *Akkermansia* and *Roseburiain* in colitis in mouse (Bu et al., 2021; Li et al., 2021), *Adlercreutzia* in influenza virus-infected mouse (Lu et al., 2021), *Faecalibacterium* in pre-eclampsia people (Chen et al., 2020), *Methanosphaera* in sheep without treatment of anthelmintic (Moon et al., 2021), Slackia in Vogt-Koyanagi-Harada patients (Li et al., 2022a; Li et al., 2022b), *Gemmiger* in immune-mediated inflammatory people (Forbes et al., 2018), and *Dorea* in HIV patients (Xu et al., 2021). Those deficient genera in *Cryptosporidium-*infected animals may be the reason for diarrhea in yaks. The genus of *Cupriavidus* was related to mycotoxin biodegradation (AL-Nussairawi et al., 2020), and the dropped *Cupriavidus* in yaks may affect mycotoxin metabolism in yaks. The previous study uncovered probiotics of *Dietzia* as a new therapy for Crohn’s disease (Click, 2015), and *Blautia*, *Phascolarctobacterium*, and *Bifidobacterium* are probiotic genera (Papizadeh et al., 2017; Chen et al., 2021; Liu et al., 2021), which demonstrated that *Cryptosporidium* led diarrhea may be due to the decrease of probiotics in the microbiota.

The shifted intestine microflora also changed their functions, as 235 significantly different enzymes were found between healthy and infected yaks (*p*<0.001) (**Figure 6A**). Only one obvious different MetaCys pathway of pentose phosphate pathway (non-oxidative branch) was found between the two yak groups (**Figure 6B**). Also, KEGG L3 analysis discovered that the abundance of 92 pathways was significantly different between healthy and infected animals (**Figure 7C**). Those results may infer that *Cryptosporidium* broke the balance of gut microbiota, which affected the microbiota function and caused diarrhea in yaks.

In the present study, significantly lower concentrations of SCFAs were found in *Cryptosporidium-*infected animals (**Figure 8**), consistent with yak diarrhea (Li et al., 2022a), LPS-induced piglets (Yang et al., 2021), and dextran sulfate sodium-induced colitis in mouse (Xu et al., 2020). SCFAs play very important roles in host physiology and energy homeostasis (Chambers et al., 2018). Among them, acetate and propionate can provide energy to peripheral tissues (den Besten et al., 2013). A previous study reported that acetate was responsible for maintaining intestine barrier integrity by inhibiting pathogens infection (Skonieczna-Żydecka et al., 2018). In a recent study, it was found that acetate could regulate IgA reactivity (Takeuchi et al., 2021), and propionate contributed to intestinal epithelial turnover and repair (Bilotta et al., 2021). Butyrate is highly related to intestine structure, energy providing to epithelial cells, and regulates immune function (Abdalkareem Jasim et al., 2022). Isobutyric acid and isovaleric acid may be related to mucosal and inflammation responses (Li et al., 2022a). Therefore, the decreased SCFAs in *Cryptosporidium-*infected yaks might have affected the intestinal barrier and immunity of the host (Aho et al., 2021), which potentially caused diarrhea in plateau yaks.

In conclusion, *Cryptosporidium* is an important zoonotic protozoon causing severe diarrhea in young animals; however, limited treatment measures are available. Here we reveal that *Cryptosporidium* infection causes dysbiosis and results in reduced SCFAs in yaks with severe diarrhea, which may give new insights regarding the prevention and treatment of diarrhea in livestock. The low sample size remains the limitation of our study.

## Data availability statement

The datasets presented in this study can be found in online repositories. The names of the repository/repositories and accession number(s) can be found below: https://www.ncbi.nlm.nih.gov/, PRJNA880359.

## Ethics statement

The animal study was reviewed and approved by ethics committee of Nanjing Agricultural University.

## Author contributions

KL and QW, research idea and methodology. HD, XC, XZ, and CZ, reagents, materials, and analysis tools. KL, writing-original draft and preparation. KM, MF-E-A, ZB, QW, JZ, SN, and KL, writing-review and editing. KL, JZ, and QW, visualization and supervision. All authors contributed to the article and approved the submitted version.

## References

[B1] Abdalkareem Jasim S. Jade Catalan Opulencia M. Alexis Ramírez-Coronel A. Kamal Abdelbasset W. Hasan Abed M. Markov A.. (2022). The emerging role of microbiota-derived short-chain fatty acids in immunometabolism. Int. IMMUNOPHARMACOL 110, 108983. doi: 10.1016/j.intimp.2022.108983 35750016

[B2] Aho V. T. E. Houser M. C. Pereira P. A. B. Chang J. Rudi K. Paulin L.. (2021). Relationships of gut microbiota, short-chain fatty acids, inflammation, and the gut barrier in parkinson’s disease. Mol. Neurodegener. 16. doi: 10.1186/s13024-021-00427-6 PMC7869249 33557896

[B3] AL-Nussairawi M. Risa A. Garai E. Varga E. Szabó I. Csenki-Bakos Z.. (2020). Mycotoxin biodegradation ability of the cupriavidus genus. Curr. Microbiol. 77, 2430–2440. doi: 10.1007/s00284-020-02063-7 32504322 PMC7415022

[B4] Amorim A. M. Nascimento J. D. (2017). Acinetobacter: an underrated foodborne pathogen? J. Infect. Dev. Ctries 11, 111–114. doi: 10.3855/jidc.8418 28248670

[B5] Bachem A. Makhlouf C. Binger K. J. de Souza D. P. Tull D. Hochheiser K.. (2019). Microbiota-derived short-chain fatty acids promote the memory potential of antigen-activated CD8+ T cells. IMMUNITY 51, 285–297. doi: 10.1016/j.immuni.2019.06.002 31272808

[B6] Berry D. Reinisch W. (2013). Intestinal microbiota: A source of novel biomarkers in inflammatory bowel diseases? Best Pract. Res. Clin. Gastroenterol. 27, 47–58. doi: 10.1016/j.bpg.2013.03.005 23768552

[B7] Bilotta A. J. Ma C. Yang W. Yu Y. Yu Y. Zhao X.. (2021). Propionate enhances cell speed and persistence to promote intestinal epithelial turnover and repair. Cell Mol. Gastroenterol. Hepatol. 11, 1023–1044. doi: 10.1016/j.jcmgh.2020.11.011 33238220 PMC7898181

[B8] Bokulich N. A. Kaehler B. D. Rideout J. R. Dillon M. Bolyen E. Knight R.. (2018). Optimizing taxonomic classification of marker-gene amplicon sequences with QIIME 2’s q2-feature-classifier plugin. MICROBIOME 6. doi: 10.1186/s40168-018-0470-z PMC5956843 29773078

[B9] Bu F. Ding Y. Chen T. Wang Q. Wang R. Zhou J.. (2021). Total flavone of abelmoschus manihot improves colitis by promoting the growth of akkermansia in mice. Sci. REP-UK 11. doi: 10.1038/s41598-021-00070-7 PMC8531128 34675239

[B10] Callahan B. J. McMurdie P. J. Rosen M. J. Han A. W. Johnson A. J. A. Holmes S. P. (2016). DADA2: High-resolution sample inference from illumina amplicon data. Nat. Methods 13, 581–583. doi: 10.1038/nmeth.3869 27214047 PMC4927377

[B11] Chambers E. S. Preston T. Frost G. Morrison D. J. (2018). Role of gut microbiota-generated short-chain fatty acids in metabolic and cardiovascular health. Curr. Nutr. Rep. 7, 198–206. doi: 10.1007/s13668-018-0248-8 30264354 PMC6244749

[B12] Chappell C. L. Darkoh C. Shimmin L. Farhana N. Kim D. Okhuysen P. C.. (2016). Fecal indole as a biomarker of susceptibility to cryptosporidium infection. Infect. Immun. 84, 2299–2306. doi: 10.1128/IAI.00336-16 27245413 PMC4962629

[B13] Chen G. Hu P. Xu Z. Peng C. Wang Y. Wan X.. (2021). The beneficial or detrimental fluoride to gut microbiota depends on its dosages. Ecotoxicol Environ. Saf. 209, 111732. doi: 10.1016/j.ecoenv.2020.111732 33373928

[B14] Chen X. Li P. Liu M. Zheng H. He Y. Chen M.. (2020). Gut dysbiosis induces the development of pre-eclampsia through bacterial translocation. GUT 69, 513–522. doi: 10.1136/gutjnl-2019-319101 31900289

[B15] Chen X. Saeed N. M. Ding J. Dong H. Kulyar M. F. Bhutta Z. A.. (2022). Molecular epidemiological investigation of cryptosporidium sp., giardia duodenalis, enterocytozoon bieneusi and blastocystis sp. Infection Free-ranged Yaks Tibetan Pigs Plateau. Pak Vet. J. 2022, 533–539. doi: 10.29261/pakvetj/2022.060

[B16] Cheng H. Ao S. Yun L. Weihong S. Hong L. Jianbo L.. (2022). RNA-Seq transcriptome analysis to unravel the gene expression profile of ovarian development in xiangxi cattle. Pak Vet. J. 42, 222–228. doi: 10.29261/pakvetj/2022.004

[B17] Click R. E. (2015). Crohn's disease therapy with dietzia: the end of anti-inflammatory drugs. Future Microbiol. 10, 147–150. doi: 10.2217/fmb.14.133 25689526

[B18] Cui Y. Chen X. Yue H. Tang C. (2022). First detection and genomic characterization of bovine norovirus from yak. Pathogens 11, 192. doi: 10.3390/pathogens11020192 35215135 PMC8874446

[B19] Dai X. Paula E. M. Lelis A. L. J. Silva L. G. Brandao V. L. N. Monteiro H. F.. (2019). Effects of lipopolysaccharide dosing on bacterial community composition and fermentation in a dual-flow continuous culture system. J. DAIRY Sci. 102, 334–350. doi: 10.3168/jds.2018-14807 30343924

[B20] den Besten G. van Eunen K. Groen A. K. Venema K. Reijngoud D. Bakker B. M. (2013). The role of short-chain fatty acids in the interplay between diet, gut microbiota, and host energy metabolism. J. Lipid Res. 54, 2325–2340. doi: 10.1194/jlr.R036012 23821742 PMC3735932

[B21] Diao N. Gong Q. Li J. Zhao D. Li D. Zhao B.. (2020). Prevalence of bovine viral diarrhea virus (BVDV) in yaks between 1987 and 2019 in mainland China: A systematic review and meta-analysis. Microb. PATHOGENESIS 144, 104185. doi: 10.1016/j.micpath.2020.104185 32272215

[B22] Du X. Liu J. Xue Y. Kong X. Lv C. Li Z.. (2021). Alteration of gut microbial profile in patients with diabetic nephropathy. ENDOCRINE 73, 71–84. doi: 10.1007/s12020-021-02721-1 33905112

[B23] Forbes J. D. Chen C. Knox N. C. Marrie R. El-Gabalawy H. de Kievit T.. (2018). A comparative study of the gut microbiota in immune-mediated inflammatory diseases–does a common dysbiosis exist? MICROBIOME 6. doi: 10.1186/s40168-018-0603-4 PMC6292067 30545401

[B24] Garrido V. Migura-García L. Gaitán I. Arrieta-Gisasola A. Martínez-Ballesteros I. Fraile L.. (2021). Prevalence of salmonella in free-range pigs: Risk factors and intestinal microbiota composition. Foods 10, 1410. doi: 10.3390/foods10061410 34207083 PMC8235412

[B25] Gryaznova M. V. Solodskikh S. A. Panevina A. V. Syromyatnikov M. Y. Dvoretskaya Y. D. Sviridova T. N.. (2021). Study of microbiome changes in patients with ulcerative colitis in the central European part of Russia. Heliyon 7, e6432. doi: 10.1016/j.heliyon.2021.e06432 PMC7970149 33748490

[B26] Han Z. Li K. Shahzad M. Zhang H. Luo H. Qiu G.. (2017). Analysis of the intestinal microbial community in healthy and diarrheal perinatal yaks by high-throughput sequencing. Microb. PATHOGENESIS 111, 60–70. doi: 10.1016/j.micpath.2017.08.025 28823792

[B27] Hou M. Xu G. Ran M. Luo W. Wang H. (2021). APOE-ϵ4 carrier status and gut microbiota dysbiosis in patients with Alzheimer disease. Front. NEUROSCI-SWITZ 15. doi: 10.3389/fnins.2021.619051 PMC7959830 33732104

[B28] Hsu Y. Chen C. Lin Y. Wu W. Chang L. Lai C.. (2019). Evaluation and optimization of sample handling methods for quantification of short-chain fatty acids in human fecal samples by GC–MS. J. Proteome Res. 5, 1948–1957. doi: 10.1021/acs.jproteome.8b00536 30895795

[B29] Kandeel M. Akhtar T. Zaheer T. Ahmad S. Ashraf U. Omar M. (2022). Anti-parasitic applications of nanoparticles: A review. PAK Vet. J. 42, 135–140. doi: 10.29261/pakvetj/2022.040

[B30] Kim S. Yu D. H. Jung S. Kang J. Park K. Chae J. B.. (2021). Biological factors associated with infectious diarrhea in calves. PAK Vet. J. 41, 531–537. doi: 10.29261/pakvetj/2021.078

[B31] Lan Y. Li K. Mehmood K. (2021). Molecular investigation of important protozoal infections in yaks. PAK Vet. J. 41 (4), 557–561. doi: 10.29261/pakvetj/2020.048

[B32] Langille M. G. I. Zaneveld J. Caporaso J. G. McDonald D. Knights D. Reyes J. A.. (2013). Predictive functional profiling of microbial communities using 16S rRNA marker gene sequences. Nat. Biotechnol. 31, 814–821. doi: 10.1038/nbt.2676 23975157 PMC3819121

[B33] Li B. Du P. Du Y. Zhao D. Cai Y. Yang Q.. (2021). Luteolin alleviates inflammation and modulates gut microbiota in ulcerative colitis rats. Life Sci. 269, 119008. doi: 10.1016/j.lfs.2020.119008 33434535

[B34] Li K. Li Z. Zeng Z. Li A. Mehmood K. Shahzad M.. (2020). Prevalence and molecular characterization of cryptosporidium spp. in yaks (Bos grunniens) in naqu, China. Microb. Pathog. 144, 104190. doi: 10.1016/j.micpath.2020.104190 32272216

[B35] Li K. Nader S. M. Zhang X. Ray B. C. Kim C. Y. Das A.. (2019a). Novel lactate dehydrogenase inhibitors with *in vivo* efficacy against cryptosporidium parvum. PloS Pathog. 15, e1007953. doi: 10.1371/journal.ppat.1007953 31356619 PMC6687188

[B36] Li N. Wang R. Cai M. Jiang W. Feng Y. Xiao L. (2019b). Outbreak of cryptosporidiosis due to cryptosporidium parvum subtype IIdA19G1 in neonatal calves on a dairy farm in China. Int. J. Parasitol. 49, 569–577. doi: 10.1016/j.ijpara.2019.02.006 31071320 PMC7089608

[B37] Li M. Yang L. Cao J. Liu T. Liu X. (2022b). Enriched and decreased intestinal microbes in active VKH patients. Invest. Ophthalmol. Vis. Sci. 63, 21. doi: 10.1167/iovs.63.2.21 PMC8842635 35142786

[B38] Li K. Zeng Z. Liu J. Pei L. Wang Y. Li A.. (2022a). Effects of short-chain fatty acid modulation on potentially diarrhea-causing pathogens in yaks through metagenomic sequencing. Front. Cell Infect. MI 12. doi: 10.3389/fcimb.2022.805481 PMC8983862 35402298

[B39] Liu X. Mao B. Gu J. Wu J. Cui S. Wang G.. (2021). Blautia-a new functional genus with potential probiotic properties? Gut Microbes 13, 1–21. doi: 10.1080/19490976.2021.1875796 PMC7872077 33525961

[B40] Love M. I. Huber W. Anders S. (2014). Moderated estimation of fold change and dispersion for RNA-seq data with DESeq2. Genome Biol. 15. doi: 10.1186/s13059-014-0550-8 PMC4302049 25516281

[B41] Lu W. Fang Z. Liu X. Li L. Zhang P. Zhao J.. (2021). The potential role of probiotics in protection against influenza a virus infection in mice. Foods 10, 902. doi: 10.3390/foods10040902 33924002 PMC8073107

[B42] Mamuad L. L. Seo B. J. Faruk M. S. A. Espiritu H. M. Jin S. J. Kim W.. (2020). Treponema spp., the dominant pathogen in the lesion of bovine digital dermatitis and its characterization in dairy cattle. Vet. Microbiol. 245, 108696. doi: 10.1016/j.vetmic.2020.108696 32456812

[B43] Mandal S. Van Treuren W. White R. A. Eggesbø M. Knight R. Peddada S. D. (2015). Analysis of composition of microbiomes: a novel method for studying microbial composition. Microbial Ecol. Health Dis. 26, 27663. doi: 10.3402/mehd.v26.27663 PMC4450248 26028277

[B44] Martinez-Lopez Y. E. Esquivel-Hernandez D. A. Sanchez-Castaneda J. P. Neri-Rosario D. Guardado-Mendoza R. Resendis-Antonio O. (2022). Type 2 diabetes, gut microbiome, and systems biology: A novel perspective for a new era. Gut Microbes 14, 2111952. doi: 10.1080/19490976.2022.2111952 36004400 PMC9423831

[B45] Meganck V. Hoflack G. Opsomer G. (2014). Advances in prevention and therapy of neonatal dairy calf diarrhoea: a systematical review with emphasis on colostrum management and fluid therapy. Acta Vet. Scand. 56, 75. doi: 10.1186/s13028-014-0075-x 25431305 PMC4246539

[B46] Mei Q. Fu Y. Huang Z. Yin N. Wang R. Xu B.. (2022). Intestinal TLR4 deletion exacerbates acute pancreatitis through gut microbiota dysbiosis and paneth cells deficiency. Gut Microbes 14. doi: 10.1080/19490976.2022.2112882 PMC9397436 35982604

[B47] Moon C. D. Carvalho L. Kirk M. R. McCulloch A. F. Kittelmann S. Young W.. (2021). Effects of long-acting, broad spectra anthelmintic treatments on the rumen microbial community compositions of grazing sheep. Sci. REP-UK 11. doi: 10.1038/s41598-021-82815-y PMC7884727 33589656

[B48] Papizadeh M. Rohani M. Nahrevanian H. Javadi A. Pourshafie M. R. (2017). Probiotic characters of bifidobacterium and lactobacillus are a result of the ongoing gene acquisition and genome minimization evolutionary trends. Microb. PATHOGENESIS 111, 118–131. doi: 10.1016/j.micpath.2017.08.021 28826768

[B49] Polanco N. Manzi L. (2008). Oxigenic effect of acinetobacter baumanniiisolated from children with acute diarrhea. Invest. Clin. 49, 59–67. 18524332

[B50] Ray K. J. Cotter S. Y. Arzika A. M. Kim J. Boubacar N. Zhou Z.. (2019). High-throughput sequencing of pooled samples to determine community-level microbiome diversity. Ann. Epidemiol. 39, 63–68. doi: 10.1016/j.annepidem.2019.09.002 31635933 PMC6996001

[B51] Salazar N. Ponce-Alonso M. Garriga M. Sanchez-Carrillo S. Hernandez-Barranco A. M. Redruello B.. (2022). Fecal metabolome and bacterial composition in severe obesity: Impact of diet and bariatric surgery. Gut Microbes 14, 2106102. doi: 10.1080/19490976.2022.2106102 35903014 PMC9341356

[B52] Sanchez Ramos L. Rodloff A. C. (2018). Identification of clostridium species using the VITEK® MS. ANAEROBE 54, 217–223. doi: 10.1016/j.anaerobe.2018.01.007 29391258

[B53] Segata N. Izard J. Waldron L. Gevers D. Miropolsky L. Garrett W. S.. (2011). Metagenomic biomarker discovery and explanation. Genome Biol. 12, R60. doi: 10.1186/gb-2011-12-6-r60 21702898 PMC3218848

[B54] Shen H. Zhu J. Ye F. Xu D. Fang L. Yang J.. (2021). Biliary microbial structure of gallstone patients with a history of endoscopic sphincterotomy surgery. Front. Cell Infect. MI 10. doi: 10.3389/fcimb.2020.594778 PMC7873689 33585269

[B55] Skonieczna-Żydecka K. Grochans E. Maciejewska D. Szkup M. Schneider-Matyka D. Jurczak A.. (2018). Faecal short chain fatty acids profile is changed in polish depressive women. NUTRIENTS 10, 1939. doi: 10.3390/nu10121939 30544489 PMC6316414

[B56] Smith R. P. Clifton-Hadley F. A. Cheney T. Giles M. (2014). Prevalence and molecular typing of cryptosporidium in dairy cattle in England and Wales and examination of potential on-farm transmission routes. Vet. Parasitol. 204, 111–119. doi: 10.1016/j.vetpar.2014.05.022 24909077 PMC7115801

[B57] Smulski S. Turlewicz-Podbielska H. Wylandowska A. Włodarek J. (2020). Non-antibiotic possibilities in prevention and treatment of calf diarrhoea. J. Vet. Res. 64, 119–126. doi: 10.2478/jvetres-2020-0002 32258808 PMC7105995

[B58] Takeuchi T. Miyauchi E. Kanaya T. Kato T. Nakanishi Y. Watanabe T.. (2021). Acetate differentially regulates IgA reactivity to commensal bacteria. NATURE 595, 560–564. doi: 10.1038/s41586-021-03727-5 34262176

[B59] Tong X. Su F. Xu X. Xu H. Yang T. Xu Q.. (2019). Alterations to the lung microbiome in idiopathic pulmonary fibrosis patients. Front. Cell Infect. MI 9. doi: 10.3389/fcimb.2019.00149 PMC6536613 31165050

[B60] Vazquez-Baeza Y. Pirrung M. Gonzalez A. Knight R. (2013). EMPeror: a tool for visualizing high-throughput microbial community data. GIGASCIENCE 2, 16. doi: 10.1186/2047-217X-2-16 24280061 PMC4076506

[B61] Wang Y. Li X. Chen X. Kulyar M. F. Duan K. Li H.. (2022). Gut fungal microbiome responses to natural cryptosporidium infection in horses. Front. Microbiol. 13. doi: 10.3389/fmicb.2022.877280 PMC9298756 35875530

[B62] Wang W. Zhai S. Xia Y. Wang H. Ruan D. Zhou T.. (2019). Ochratoxin a induces liver inflammation: involvement of intestinal microbiota. MICROBIOME 7. doi: 10.1186/s40168-019-0761-z PMC6883682 31779704

[B63] Wei X. Wang W. Dong Z. Cheng F. Zhou X. Li B.. (2021). Detection of infectious agents causing neonatal calf diarrhea on two Large dairy farms in yangxin county, Shandong province, China. Front. Veterinary Sci. 7. doi: 10.3389/fvets.2020.589126 PMC7892430 33614754

[B64] Wu I. Lin C. Chang L. Lee C. Chiu C. Hsu H.. (2020). Gut microbiota as diagnostic tools for mirroring disease progression and circulating nephrotoxin levels in chronic kidney disease: Discovery and validation study. Int. J. Biol. Sci. 16, 420–434. doi: 10.7150/ijbs.37421 32015679 PMC6990903

[B65] Xu H. Ou Z. Zhou Y. Li Y. Huang H. Zhao H.. (2021). Intestinal mucosal microbiota composition of patients with acquired immune deficiency syndrome in guangzhou, China. Exp. Ther. Med. 21, 391. doi: 10.3892/etm.2021.9822 33680113 PMC7918403

[B66] Xu Z. Tang H. Huang F. Qiao Z. Wang X. Yang C.. (2020). Algal oil rich in n-3 PUFA alleviates DSS-induced colitis *via* regulation of gut microbiota and restoration of intestinal barrier. Front. Microbiol. 11, 615404. doi: 10.3389/fmicb.2020.615404 33391246 PMC7772400

[B67] Yang Q. Huang X. Zhao S. Sun W. Yan Z. Wang P.. (2017). Structure and function of the fecal microbiota in diarrheic neonatal piglets. Front. Microbiol. 8. doi: 10.3389/fmicb.2017.00502 PMC5364137 28392784

[B68] Yang C. Wang M. Tang X. Yang H. Li F. Wang Y.. (2021). Effect of dietary Amylose/Amylopectin ratio on intestinal health and cecal microbes’ profiles of weaned pigs undergoing feed transition or challenged with escherichia coli lipopolysaccharide. Front. Microbiol. 12. doi: 10.3389/fmicb.2021.693839 PMC8329381 34354689

[B69] Zeineldin M. Aldridge B. Lowe J. (2018). Dysbiosis of the fecal microbiota in feedlot cattle with hemorrhagic diarrhea. Microb. PATHOGENESIS 115, 123–130. doi: 10.1016/j.micpath.2017.12.059 29275129

[B70] Zhai H. Luo Y. Ren W. Schyns G. Guggenbuhl P. (2020). The effects of benzoic acid and essential oils on growth performance, nutrient digestibility, and colonic microbiota in nursery pigs. Anim. FEED Sci. TECH 262, 114426. doi: 10.1016/j.anifeedsci.2020.114426

[B71] Zhang X. Li C. Cao W. Zhang Z. (2021). Alterations of gastric microbiota in gastric cancer and precancerous stages. Front. Cell Infect. MI 11. doi: 10.3389/fcimb.2021.559148 PMC7966516 33747975

[B72] Zhang S. Wang H. Zhu M. (2019). A sensitive GC/MS detection method for analyzing microbial metabolites short chain fatty acids in fecal and serum samples. TALANTA 196, 249–254. doi: 10.1016/j.talanta.2018.12.049 30683360

